# Early Intervention and Nonpharmacological Therapy of Myopia in Young Adults

**DOI:** 10.1155/2018/4680603

**Published:** 2018-02-08

**Authors:** Katarzyna Zorena, Aleksandra Gładysiak, Daniel Ślęzak

**Affiliations:** ^1^Department of Immunobiology and Environment Microbiology, Medical University of Gdańsk, Gdańsk, Poland; ^2^Students' Scientific Group Department of Immunobiology and Environment Microbiology, Medical University of Gdańsk, Gdańsk, Poland; ^3^Emergency Medicine Workshop, Department of Emergency Medicine, Medical University of Gdańsk, Gdańsk, Poland

## Abstract

Myopia is a condition of the eye where parallel rays focus in front of, instead of on, the retina, which results in excessive refractive power of the cornea or the lens or eyeball elongation. Studies carried out in recent years show that the etiology of myopia is complex with genetic and environmental factors playing a role. Refraction defects decrease the quality of vision, while progressing myopia can lead to partial loss of vision, which can be particularly dramatic in young adults. Therefore, it is so crucial to take appropriate actions aimed at preventing myopia progression. This is a review of nonpharmacological therapeutic possibilities of refraction defect prevention in young adults, with special regard to myofascial therapy, osteopathy, and massage of acupuncture points surrounding the eye.

## 1. Introduction

Myopia, being defined as more than or equal to −0.50 diopter (D), is one of the most commonly occurring defects of refraction. The prevalence of myopia varies in different parts of the world, with the fastest growing tendency in the countries of East Asia, especially in Singapore and China [1]. In studies carried out with a participation of 12-year-olds, myopia was observed in as much as 62% of children examined in Singapore and 49.7% in China, compared to 20.0% in the USA or 11.9% in Australia [2, 3]. Studies conducted in Poland on a large number children aged 6–18 years showed that among 11-year-old children, the prevalence of myopia amounts to 12.17% [4]. Moreover, it was observed that there is a relation between reading and writing from a short distance and the development of myopia. Computer work leads to it as well while watching TV was not found to cause the development of myopia. The authors also observed that outdoor activity leads to lower prevalence of myopia in children and adolescents in the Polish population [5].

**Figure 1:**
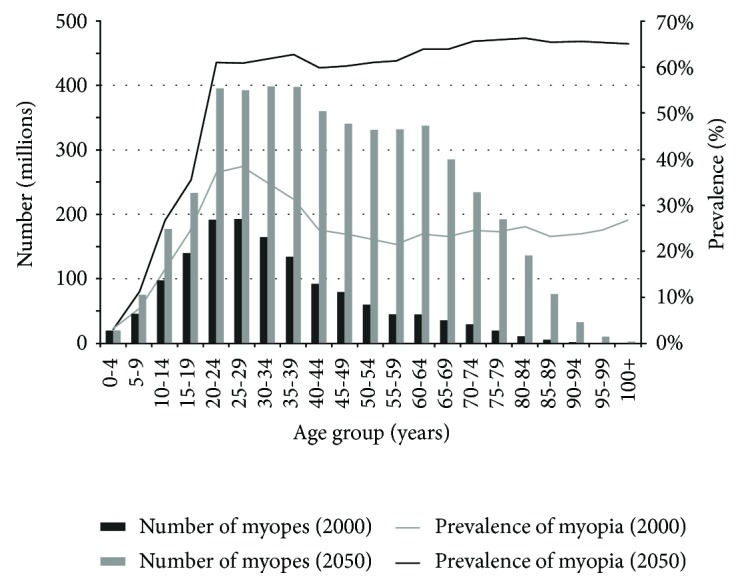
Graph showing the likelihood of developing myopia in people aged between 10 and 79 years [3].

Myopia is divided into three levels: low (≤−3.0 D), medium (between −3.0 D and −6.0 D), and high (over −6.0 D). Approximately one-fifth of people with myopia developed high myopia (≥−6 D), which can result in loss of vision due to retinal detachment, neovascularization, cataract, glaucoma, or macular atrophy. High axial myopia is caused by a too long eyeball and progresses with its elongation and stretching in the posterior pole of the sclera, choroid, and retina [6, 7]. Recent studies showed that in 2000, the number of people that developed myopia amounted to 1406 million while in 2010, it grew to 1950 million. It is predicted that by 2050, this number will have increased to 4758 million [3]. Moreover, in the same manuscript, the authors pointed out that myopia is a condition occurring in people aged 10–39 years. However, the authors suggest that by 2050, myopia will be developing in patients aged 10–79 years (Figure 1) [3].

The etiology of myopia is complex with genetic and environmental factors playing a role, for example, education and urbanization, lifestyle, vitamin D, and electronic devices including smartphones [8–13]. Furthermore, the authors point out that myopia varies among different ethnic groups; that is, it is more common among children born in East Asia than those born in European ethnic groups [9]. Studies indicated that myopia occurs in 33–60% of children, whose parents also developed myopia. If only one parent presents with myopia, the defect occurs in 23–40% of children. In the case of children whose parents do not have myopia, the occurrence amounts to 6–15% [8].

## 2. Risk Factors for Myopia in Young Adults

### 2.1. Genetic Factors versus the Risk of Myopia Development

It is believed that inheritance of the refraction defect is multifactorial and of polygenic nature. There is extensive research conducted, aimed at identifying the locus in the human genome that is responsible for myopia and at assessing the effect of the genome on the phenotype presentation, that is, the status of the refraction defect. So far, there have been approximately 30 loci identified that are associated with myopia, among which at least 13 are associated with high myopia [6, 10, 11]. The list of genes potentially meaningful for the development of both the hereditary and the sporadic form of high myopia, located in the identified loci, is long and goes beyond the scope of this article. The list includes candidate genes such as *TGIF* (MYP2), *LUM* (MYP3), *COL1A1* (MYP5), IGF-1, *PAX6* and *SOX2*, *BICC1*, and *RASGRF1.* However, in subsequent years, there were contradictory study results published, which did not fully confirm the effect of the above-mentioned genes in the development of myopia [11–13]. A recent study indicated that genetic variants in BICC1 and RASGRF1 are closely associated with high myopia, which appears to be a potential candidate for high myopia in a Chinese Han population [11]. Meta-analysis showed a relation between gene PAX6 rs644242 and high myopia in the Asian population [12]. Interestingly, it has been shown that polymorphisms within vitamin D receptor (VDR) at 12q13.11 and GC at 4q12-13 appear to be associated with low-to-moderate amounts of myopia in white subjects [13]. Biological activity of vitamin D is also manifested in genome activity (together with nuclear receptors, vitamin D connects with the vitamin D receptor (VDR) and then creates a heterodimer with the retinoid X receptor (RXR) of transcription factor properties. Therefore, there are studies available, indicating that the physiological role of active metabolites of cholecalciferol (D3) is also manifested in the effect on the expression of genes that can affect myopia [13–16].

### 2.2. Vitamin D Level versus Myopia

Studies carried out to this date showed that low level of vitamin D3 observed among various populations can be associated with the increasing prevalence of myopia [17, 18]. Yazar et al. suggested that lower vitamin D3 level is one of the factors that mediate in the prevalence of myopia between various ethnic groups [17]. In many populations, the main source of vitamin D3 is the endogenous synthesis triggered by the exposure of the skin to sunlight. Hypovitaminosis D is common among many populations, and its level is constantly decreasing. This probably results from behavioral changes which lead to the limited exposure to sunlight. Fast growth in the prevalence of myopia in East Asian populations results from limitations in the amount of time people spend outside in the open air. Within the last 10 years, many observational studies confirmed the hypothesis that longer time spent in the open air protects one against myopia [5, 18–21]. It has been shown that increased UVB exposure in children and adolescents is associated with regression of myopia, especially during the puberty period [20]. Pan et al. analyzed studies published in Great Britain, which pertained to the relation between the time spent outdoors, the level of vitamin D, and myopia. They concluded that the time spent outdoors decreases the risk of myopia development, which was well established in numerous observational studies. They identified 5 studies showing the relation between vitamin D level in blood and the risk of myopia and 2 studies investigating changes in vitamin D receptor as a potential risk factor for the development of myopia. Most of the proof was obtained from large-scale studies. Proof confirming that vitamin D plays any role in the development of myopia was too indefinite, and the mechanisms behind it were vague. Currently, it is still unclear whether vitamin D level triggers the onset and promoted the progression of myopia. According to Pan et al., the level of vitamin D can be used only as a marker of exposure to the external environment, which is an actual protective factor against myopia [21].

### 2.3. Lifestyle and Myopia in Young Adults

As early as in the 80s, near work was already considered a key risk factor for developing myopia. In subsequent years, the authors analyzed the effect of the number of near work hours on the occurrence of myopia in children. They observed that myopia was statistically more common in children spending more than 0.8 hour in front of a computer screen and reading and/or writing for over 2 hours a day [22]. In a 4-year-long study, Guo et al. found a relation between shorter time spent outside, elongation of time spent in closed spaces when learning, and axial elongation of the eyeball, resulting in the progression of myopia. The above-mentioned studies also confirmed the effect of other factors such as the child's sex, place of residence, and the level of parents' education. The authors concluded that exposure to daylight prevents axial elongation of the eyeball [22]. Another study, conducted on a group of 514 children, confirmed that myopia in parents and less time spent on physical activity are significant risk factors for the development of myopia in children [23]. According to the authors, devoting additional 10 hours per week to reading increases the risk of myopia progression by −0.08 diopters [24]. Studies carried out in Poland showed a double increase in the occurrence of myopia among students at medical universities compared to people of the same age but not studying. It is believed that approx. 13% of medical university students have high risk of developing myopia during their studies, while those who already developed this condition have high risk of progression [25]. The most recent study shows the low occurrence of myopia among farmers, which confirms the positive effect of spending time in the open air on the prevention of myopia. Studies also indicate that the incidence of myopia is similar in children whose parents were farmers and those whose parent were not farmers. Thus, it can be concluded that the distribution of genes responsible for myopia is similar in both groups. Very low incidence of myopia among farmers results from the fact that they have no risk factors. Also, it can be concluded that in the case of no environmental risk factors, myopia will not develop, even with genetic load [26].

### 2.4. The Effect of Electronic Devices on Myopia

Recent studies have shown that looking at and reading text in a small font on a smartphone lead to eye strain, blurred vision, dizziness, and eye dryness. Blurred vision and tension of neck muscles can cause headaches [27–31]. Moreover, in recent years, the technology of head-mounted displays (HMD) was developed, which allows the users to experience virtual reality (VR) [27, 28]. The HMD holds the phone at a certain distance from the lenses, close to the eyes. People using a HMD suffer from visual discomfort (headaches, blurred vision) and eye strain. Han et al. carried out a subjective and objective analysis of the visual discomfort and eye strain caused by the use of HMD and smartphones [28]. The experiment showed that the use of both HMD and smartphones may cause myopia, even though the users were not able to see the loss of the quality of vision. This mild change is temporary, and it subsides within a few minutes. Results show that during the use of HMD, the user presents with visual discomfort and eye strain, while during the use of smartphones, the intensity of symptoms was statistically insignificant. HMD did not cause any significant eye strain, but it did cause eye dryness, which can lead to an eye infection [28]. Since introduction of smartphones into the market in 1997, the number of patients with progressive myopia has increased by 35%. Smartphones are currently omnipresent, and they accompany both children and adults in their everyday life. The mean time of daily smartphone use is constantly growing, and between 2011 and 2013, it almost doubled from 98 minutes to 195 minutes [29]. National studies conducted in 2013 in the USA showed that 39% of the Americans use their cell phones in the bedroom one hour before they go to sleep, while among adolescents, this proportion was twice as high [30]. Smartphones are frequently equipped with light-emitting diodes (LEDs), which suppress the production of melatonin, cause mood changes, affect cognitive function, and contribute to fatigue. An average smartphone or tablet user holds the device approx. 30 cm from his/her face, some even only 18 cm away from their face, compared to 40 cm when reading a newspaper. It is estimated that within the next 10 years, the problem of myopia will increase by 50% [31].

### 2.5. Relation between High Level of Intelligence and Myopia

Colom et al. defined intelligence as a general, mental ability to reason, solve problems, and learn [32]. The relation between myopia and high intelligence level was investigated by many scientists [9]. The first study carried out in 1995 showed a strong positive correlation between myopia and high intelligence [33]. A study carried out in Singapore assessed the relation between myopia and the IQ in a group of 1204 Chinese children aged 10–12 years in three Chinese schools. The analysis involved several parameters including the age, sex, type of school, family history of myopia, father's level of education, the number of books read per week, and IQ. The investigators observed that children with higher IQ develop higher myopia. What is more, the authors emphasized that the refraction defect cannot only result from the number of books read per week, and the IQ can be an independent risk factor for myopia [34]. In addition, other studies have shown that children who read a lot get higher scores in IQ tests and are more prone to myopia, because they tend to do more near work. On the other hand, the researchers revealed a statistically significant correlation between the occurrence of myopia in children and higher education of their parents [35, 36].

## 3. Early Intervention and Nonpharmacological Therapy of Myopia

### 3.1. Glasses and Contact Lenses in the Progression of Myopia

In order to eliminate myopia or hinder its progression, several therapeutic methods have been tested, with a relatively poor effect though. These included contact lenses, bifocal and multifocal spectacle lenses, and pinhole glasses. It was observed that progressive spectacle lenses do hinder the progression of myopia with statistical significance, though this effect is marginal [37, 38]. Another type of glasses recommended in myopia is the nonlensed, pinhole glasses invented thousands of years ago in India [39]. In the last 10 years, billboards have been informing us that the use of nonlensed glasses helps in exercising the oculomotor muscles and their relaxation. However, there is no sufficient proof to confirm the effect of continuous wearing of glasses on the permanent correction of ametropia (abnormal ratio of accommodation abilities and the eyeball length) and presbyopia [40]. The study enrolled 48 people aged 20–50, wearing nonlensed glasses 7 days a week, and it was observed that the visual acuity at a short distance was improved, but the patients also presented with decreased sensitivity in the field of vision test and contrast sensitivity testing [40]. Soft contact lenses do not guarantee control of myopia progression either. Hard lenses, compared to soft ones, allow for a decrease in progression by 0.63 D, but they have been reported to cause temporary morphological corneal lesions [41].

Retrospective studies and case studies carried out so far have indicated that modern orthokeratology methods allow us to hinder the progression of myopia in children [42–45]. One of the therapeutic methods to treat low and medium myopia in children and adolescents is the contact lenses of properly selected shape worn nightly. It is the so-called ortho-correction, which is based on reshaping (flattening) the anterior surface of the cornea with rigid permeable contact lenses. Flattening of the surface of the cornea sustains for the entire day after removal of the therapeutic lenses. Ortho-correction can be applied in myopia between 1 D and 5 D [44]. The study showed that orthokeratology was a therapeutic alternative which allows for effective limitation of the progression of myopia, compared to correction with glasses, in a group of girls with a slower pace of progression before the beginning of the study. Moreover, they presented with a lower level of myopia at the beginning of the study, deeper anterior chamber of the eye, higher optical power of the cornea, more elongated shape of the cornea, and larger diameter of the iris and pupil, and their parents presented with a lower level of myopia [42, 44]. It was observed that orthokeratology slows down the progression of myopia by 30–50%, which translates to 0.5 D per year, compared to correction with glasses and soft contact lenses [42]. However, during a 7-year-long observation, the authors found no statistically significant differences in respect of the change of the length of the eyeball between the group of subjects treated with orthokeratology and the control group [46]. Moreover, the safety profile of orthokeratology is a matter of concern. Compressive forces of the reverse geometry rigid lenses can disturb the corneal epithelium, and a too long overnight wear can potentially cause infectious keratitis. The previously obtained clinical data indicated that most microbiological infections associated with the use of the ortho-k lens resulted in a corneal scar and almost 10% eyes required surgical treatment [47].

### 3.2. Use of “Chinese Eye Exercises” in the Treatment of Myopia in Children

**Figure 2:**
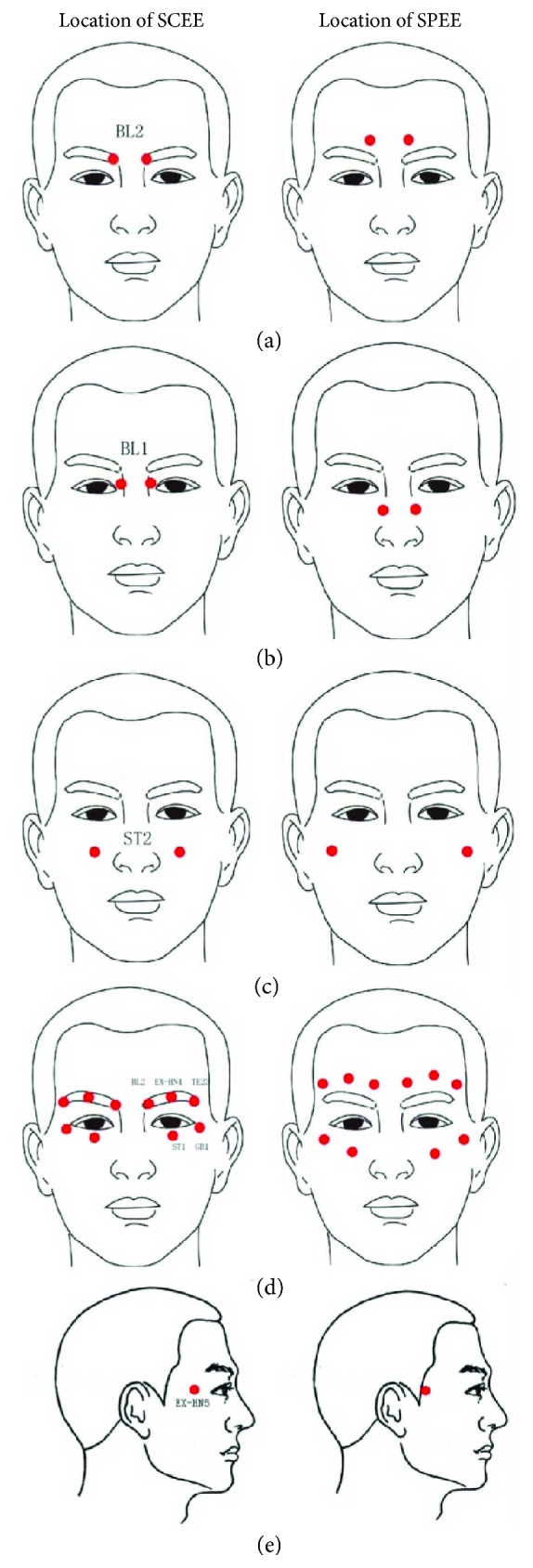
Location of the acupuncture points used in the “Chinese eye exercise” technique [49]. Acupoint code (name in Chinese)—BL-2: cuanzhu; BL-1: jingming; ST-2: sibai; EX-HN5: taiyang; TE-23: sizhukong; EX-HN4: yuyao; GB-1: tongziliao; ST-1: chengqi [49].

In 1963, the Chinese government approved Chinese eye exercises in order to prevent myopia and protect the eyesight in children. The exercises have become a common and everyday habit of children in primary and secondary schools. Chinese eye exercises are a type of massage of acupuncture points surrounding the eye, and they come from traditional Chinese medicine [48]. There are 16 acupuncture points located symmetrically within the human face. The BL-2 point is located at the medial end of the brows; BL-1 is located at the medial angle of the eye; ST-2 is located in the line of the pupil, at the level of the nostrils; EX-HN5 is located on the forehead; and TE-23, EX-HN4, GB-1, and ST-1 are located around the eye sockets (Figure 2) [49]. Points BL-2, BL-1, and ST2 must be pressed for 1 minute, while the other ones for 30 seconds [49].

According to the most recent reports, there is no relation between the Chinese eye exercises and common occurrence of myopia as indicated in some studies aimed at evaluating the effectiveness of Chinese eye exercises on delayed accommodation in children [49–51]. A significant improvement in accommodation delay (−0.10 D) directly after the exercises was observed in 54% of children. In 46% of children from the study group, there was no improvement observed in accommodation delay, which may have been caused by genetic or psychological factors, sensitivity of the acupuncture points, or the severity of the defect [49]. One hundred ninety patients participated in this study. The mean age was 12.62 ± 0.56. Sixty-three patients performed 5-minute exercises every day, before going to school. Therapy of the BL-2 and ST-1 points can trigger tear secretion, increase the level of lactoferrin, and affect the intraocular pressure. Lactoferrin is an important element of the immune system, which is probably associated with its affinity for iron. By picking up and bonding iron in the body, it prevents bacteria from having access to the ion necessary for their development and growth [52]. Chinese eye exercises can increase the flow of blood to the eyeball and improve the response of the parasympathetic nervous system of the ciliary muscle by stimulation of the area of the eye or the visual cortex, and by that, it affects the process of accommodation as indicated in the first study assessing the long-term use of Chinese eye exercise in children [52, 53]. The studies were conducted in 201 primary school students aged 12.7 ± 0.5. The students performed 5-minute-long exercises at least once a day for 2 years. In a group of those who systematically and correctly performed the exercises (15% of all participants), after 2 years, the scientists observed slightly slower myopia progression (0.15 D) than that in the participants who did not perform the exercises correctly. The limiting factor in the study was the fact that approximately 90% of the children were not able to perform the exercises in accordance with the standard procedures. It is problematic to locate the points properly and apply appropriate pressure force [54]. Other studies also showed the positive effect of eye exercises of acupoints on the myopia in children. A study by Lin et al. indicated that 10-minute eye exercises performed daily have a less protective effect in children aged 4–17 years living in rural areas [55].

### 3.3. Use of Vision Training in Patients with Myopia

The use of visual training with a simultaneous use of soft lenses in subjects with myopia is another nonpharmacological therapy of myopia in young adults. The training is to improve the accuracy and dynamics of accommodation in young adults with myopia. An interesting visual training was carried out by Allen et al. [56]. The study enrolled 94 people with myopia. The study group used contact lenses with spherical aberration (SA) and performed visual training, while the control group used contact lenses without SA and did not perform any training. The patients performed 18-minute-long exercises with the use of a flipper for 6 weeks. The training comprised 82 exercises with a flipper +2.00 D/−2.00 D, at a distance of 40 cm. After 3 months, their accommodation improved significantly. Accommodation response to near objects was improved by SA lenses, and the index of active accommodation was improved as a result of the visual training. The study confirmed that the treatment of the accommodation function is effective. Both the near and distant viewing functions were significantly improved after the training, compared to baseline [56].

### 3.4. Use of the Myofascial Therapy in the Treatment of Myopia

**Figure 3:**
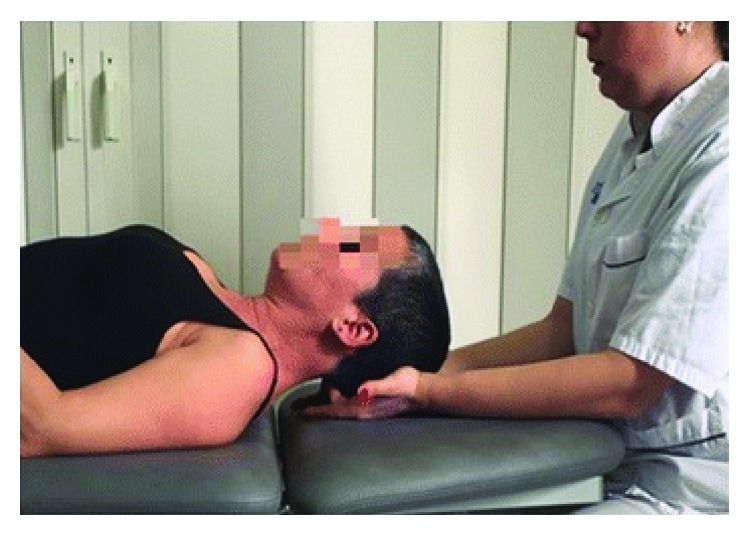
The technique relaxing the suboccipital muscles [60].

Disturbances in vision lead to increased tension within the trapezius and the sternocleidomastoid muscle, which can lead to cranial tension. Patients commonly compensate vision problems by bending forward or turning their head to the sides. Overuse of the oculomotor muscles can cause headaches and neck pain. Those with myopia commonly present with protractive positioning of the head and cervical segment of the vertebral column, which leads to increased tonus of the thoracic muscles, the descending fibres of the trapezius, the levator scapulae, and the sternocleidomastoid muscle and decreased tonus of the deep muscles that stabilize the cervical segment of the vertebral column, the rhomboid muscles, and the serratus anterior [57]. The suboccipital muscles are responsible for the stabilization of the upper cervical segment of the vertebral column and the normal movement of the cranium relative to the atlas and the atlas relative to the odontoid vertebra. One of the suboccipital muscles, the lesser posterior straight muscle of the head, has a proprioceptive function ensuring proper deep sensibility in the area of the head and neck. Atrophy of this muscle can result in decreased postural balance of the head and the neck [58]. Head protraction can be associated with upper crossed syndrome, in which deep cervical flexors are weakened and the suboccipital muscles are shortened. The upper cervical segment becomes extended, and there is compensation which is to lift the eyeballs. Long-lasting shortening of the suboccipital muscles can result in ischemia and cause complaints including headaches and dizziness, tinnitus, and nuchal rigidity [59]. Moving the head forward results in mechanical offload of the neck. Due to lack of muscular balance resulting from overloading, some muscles become weak. The technique of relaxation of the suboccipital muscles is based on putting pressure in the ventral direction in the suboccipital area while the patient's head is resting on the therapist's hands, as presented in Figure 3 [60]. The pressure should be even and constant for 5 minutes. The use of this technique is to improve the mobility of the cervical spine and restore proper positioning of the head [60].

**Figure 4:**
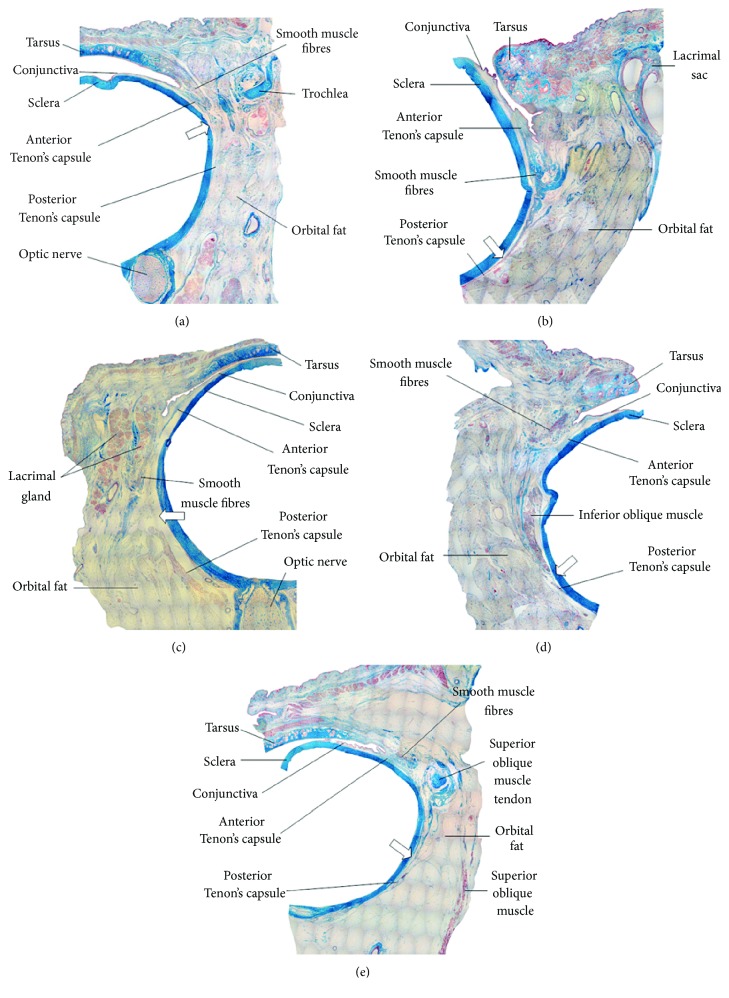
The structure of the Tenon's capsule: (a) superomedial projection; (b) inferomedial projection; (c) superolateral projection; (d) inferolateral projection [68].

Proper selection of corrective glasses is of key significance for maintaining proper tension balance within the head and neck. The use of contact lenses with a too short focal length leads to habitual lowering of the head when reading or performing near work. Placing the glasses too high or too low on the nose can result in head bending to the front or back. This mechanism can activate trigger points in the suboccipital muscles and the semispinalis muscle of the head and neck. According to Jack Holladay, most corrective glasses are too strong and lead to chronic tension within the eyes and head [57]. The myofascial trigger point (MTP) is a hypersensitive area in skeletal muscles and has a form of a taut band or a nodule. The area is painful on compression and stretching. It can cause characteristic signs such as pain, hypersensitivity to touch, disturbed motor activity, and autonomic symptoms [61]. Cachinero-Torre et al. found a relation between hypersensitivity of the supraorbital nerve and the presence of an active trigger point in the lateral rectus of the eye in persons with tension-type headaches [62]. According to the International Headache Society, tension-type headaches (TTH) are the most common type of initial headaches in all age groups [63]. Despite the high number of studies conducted in recent years, the etiological mechanism of TTH has not yet been fully established. It is presupposed that disturbances in the trochlear region, central sensitivity of the trigeminal nerve, and muscular pain are the main factors associated with TTH [64]. Moreover, the oculomotor system is closely related to the above-mentioned factors and the signs of TTH in the following ways: Firstly, disturbances in the trochlear region cause orbital pain that can stimulate oculomotor muscles and disturb their dynamics [65]. Secondly, the oculomotor muscles are innervated by the supraorbital branch of the trigeminal nerve. Consequently, disturbed function of the lateral rectus muscle can trigger the signs of TTH [66]. Thirdly, “visual effort,” that is, excessive load of the visual system in inappropriate conditions (e.g., looking at a computer screen in a dark room and from an insufficient distance), was described as the cause of disturbed mobility of the eyeball and the cause of TTH [67]. The fascia of the head and neck has a very important proprioceptive function in the human body. It frequently is a source of tension pain of the head, neck, and temporomandibular joint and a source of disturbed vision. The Tenon's fascia is a deep fascia of the eye. According to Kakizaki et al., it can be divided into three parts: anterior, central, and posterior. The central part makes a fascial sheath for four recti of the eye and two oblique muscles and a separate sheath for the elevating muscle of the upper eyelid. The posterior part, on the other hand, fuses with the sheath of the optic nerve (Figure 4) [68].

**Figure 5:**
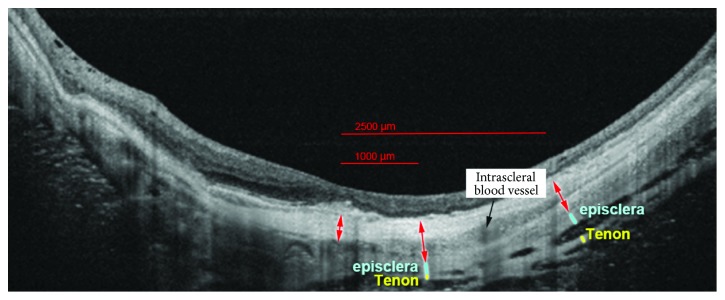
The method used to measure the diameter of the episclera, sclera, and Tenon's fascia [70].

Moreover, studies indicated that the Tenon's fascia and the muscles of the eye constitute a functional whole; therefore, it is not possible to separate the muscular activity from fascial activity. There is a close relation between each muscle and its fascial sheath. The fascia holds the muscles together and strengthens their activity. Each of them needs some preliminary tension of the Tenon's fascia and the tendon of the eye. Any tension in the myofascial system that affects the orbit is picked up by the periorbita, which is connected with the Tenon's fascia with the epicranial fascia. Constant, excessive tension coming from the limbs can be transferred in the proximal direction as far as to the head, along the connection of the trunk with the platysma muscle and neck extensors [69–71]. An interesting study was conducted by Ohno-Matsui et al. who made an attempt to investigate the structural features of the posterior part of the episclera and the Tenon's fascia in patients with high myopia [70]. The study involved 278 eyes of 175 patients aged 60.9 ± 11.4 years with a refraction defect of >−8 D or an axial length of ≥26.5 mm. The posterior parts of the episclera and the Tenon's fascia were examined with optical coherence tomography. Analysis with the use of OCT allowed for a visualization of the posterior part of the sclera and the episclera, and in some cases also of the Tenon's fascia (Figure 5) [70]. It was observed that eyes with a detectable episclera had significantly longer axial length and thinner central retinal thickness than eyes without it. Moreover, the fibres seemed to be more loosely arranged and more split up in the Tenon's capsule [70].

The diameter was measured along the axis perpendicular to the curvature of the pigmented epithelium of the retina. The red arrow indicates the sclera. The blue arrow indicates the episclera. The yellow arrow indicates the Tenon's fascia [70]. The fascia is characterized by a multilayer structure of the collagen fibres, which results in more complicated mechanisms than those involving tendons. Such orientation of the fibres ensures high resistance in case of multidirectional stretching forces [69, 72]. Experimental studies showed that the resistance of a deep fascia that is 1 cm wide exceeds 390 N. Moreover, this resistance seems to be associated with the muscular mass and maximum muscle power. This allows us to presuppose that the deep fascia works like a tendon, transferring force from one segment to another [73]. Trindade et al. showed that the deep temporal fascia plays a fundamental role in the transfer of tension and stretching forces generated by temporal muscles on the masticatory system [74]. The deep fascia of the neck consists of three laminae, and each of them is attached to the muscles below. It is not possible to separate the function of the deep fascia from the function of the extensors of the head and the cervical segment of the vertebral column. Due to the continuity along the superficial fascia and the tendinous galea, tension can be transferred from the trunk to the eyeball. There is a similar continuity along the superficial fascia of the chest and the platysma, which is connected with the superficial muscular aponeurotic system (SMAS) comprising the mimical muscles of the face [69, 72, 73].

### 3.5. Use of Osteopathy in the Treatment of Myopia

Osteopathic medicine is a noninvasive, alternative form of manual therapy, classified as a supplementary manipulative technique [75–77]. According to Sandhouse et al., osteopathic manipulative treatment decreases intraocular pressure and affects the field of vision and positioning of the eyeballs. Osteopathic techniques applied to cranial areas can have a positive effect on the visual function in young adults with myopia [76]. The authors carried out the analysis on a group of 29 young adults with myopia, aged 24.38 ± 3.03. Within the study, 15 patients were subject to one session of osteopathic manipulative treatment (OMT). After this treatment, the researchers observed improvement in visual acuity from distance and an increase in the size of the pupil [76]. There are two potential mechanisms to explain the improvement in visual acuity. Firstly, oculomotor muscles are connected with the eyeball, the orbit, and the adjoining muscles that are directly or indirectly connected with the sphenoid bone. If bones that are connected with the oculomotor muscles change their position (by cranial manipulations), the eye changes its shape and consequently its axial length. Secondly, parasympathetic innervation of the eyes is provided by the oculomotor nerve and the ocular branch of the trigeminal nerve, which run through the fissure of the sphenoid bone. Manipulations of the sphenoid bone release the bones or fascial restrictions, which can restore normal functioning of the autonomic system by decreasing the afferent activity of the oculomotor nerve and the trigeminal nerve [76, 77].

## 4. Conclusions

Myopia is currently perceived as a civilization-related disease, and sight defects in children have their onset at a younger age. There is no satisfactory effective method of prevention of refraction defects in children and adolescents. Myopia is usually treated with glasses, contact lenses, or surgical procedures. However, this kind of therapy is aimed at limiting the defect, not preventing it. Within the last decade, researchers published the results of their studies of vision training, myofascial therapy, and osteopathy in the treatment of myopia. They have suggested probable mechanisms of brain-eye paths and made another step on the way to understanding and knowing how to prevent refraction defects in young adults. Without doubt, there is a need for further research in order to identify the most effective therapy of refraction defects.

## Abbreviations

D: Diopter
BL-1: Jingming
BL-2: Cuanzhu
EX-HN4: Yuyao
EX-HN5: Taiyang
GB-1: Tongziliao
HMD: Head-mounted display
MTP: Myofascial trigger point
OCT: Optical coherence tomography
OMT: Osteopathic manipulative treatment
SA: Spherical aberration
SMAS: Superficial muscular aponeurotic system
ST-1: Chengqi
ST-2: Sibai
TE-23: Sizhukong
TTH: Tension-type headaches.
